# Laparoscopic approach for total cystectomy in treating hepatic cystic echinococcosis[Fn FN1]

**DOI:** 10.1051/parasite/2014065

**Published:** 2014-12-10

**Authors:** Haitao Li, Yingmei Shao, Tuerganaili Aji, Jinhui Zhang, Kafayat Kashif, Qinglong Ma, Bo Ran, Hao Wen

**Affiliations:** 1 Hepatobiliary & Hydatid Department, Digestive and Vascular Surgery Centre, First Affiliated Hospital of Xinjiang Medical University Urumqi 830011 PR China; 2 State Key Lab Incubation Base of Xinjiang Major Diseases Research (2010DS890294) and Xinjiang Key Laboratory of Echinococcosis, First Affiliated Hospital of Xinjiang Medical University Urumqi 830011 PR China; 3 Department of Liver and Laparoscopic Surgery, Digestive and Vascular Surgery Centre, First Affiliated Hospital of Xinjiang Medical University Urumqi 830011 PR China

**Keywords:** Cystic echinococcosis, Laparoscopic surgery, Total cystectomy

## Abstract

*Background*: The laparoscopic approach has been proposed for treating hepatic cystic echinococcosis (HCE) and has already been used in clinical practice, mostly for non-radical operations. In this study, we aimed to evaluate the feasibility of total cystectomy of HCE under laparoscopy (LS).

*Results*: A retrospective review of the medical records obtained from 22 patients diagnosed with HCE between June 2009 and June 2013 and treated with an LS approach was conducted in the First Affiliated Hospital of Xinjiang Medical University. A total of 15 patients underwent total cystectomy of HCE using LS. The average time of surgery was 174 min (160–210 min). Intraoperative bleeding was 103 mL (80–200 mL). The mean duration of hospitalization was 7 days (6–15 days). Seven patients were transferred to open surgery (OS). For these patients, the average duration of surgery was 177 min (150–230 min). Intraoperative bleeding was 237 mL (160–350 mL), and the mean duration of hospitalization was 10 days (8–15 days). The most frequent postoperative complications were hydrops in the surgical area (two cases in LS and three cases in OS), and temporary bile leakage (one patient in the LS group). Recurrence was not seen in any cases in either group with a follow-up of 6–12 months.

*Conclusions*: Total cystectomy of HCE appears to be safe and effective in selected patients with unique, small-sized, superficially located cysts. To establish precise recommendations about the technique and its indications, prospective studies are necessary.

## Abstract (fr)

*Contexte* : La voie laparoscopique, essentiellement pour des interventions non radicales, a déjà été utilisée en pratique clinique pour traiter l’échinococcose kystique hépatique (EKH). Dans cette étude, notre but était d’évaluer la faisabilité de la cystectomie totale de l’EKH sous laparoscopie (LS).

*Résultats* : L’analyse rétrospective des dossiers médicaux de 22 patients atteints d’EKH entre Juin 2009 et Juin 2013 et opérés sous laparoscopie a été réalisée au 1^er^ Hôpital Universitaire de l’Université Médicale du Xinjiang, Chine. Quinze patients ont pu avoir une cystectomie totale per-laparoscopique de leurs kystes. Le temps moyen d’intervention était de 174 minutes (160 à 210 min), le saignement per-opératoire de 103 mL (80–200 mL), et la durée moyenne d’hospitalisation de 7 jours (6–15 jours). Sept patients ont dû être laparotomisés (LT). Chez ces patients, la durée moyenne d’intervention était de 177 minutes (150–230 min); le saignement de 237 mL (160–350 mL), et la durée moyenne d’hospitalisation de 10 jours (8–15 jours). Les complications post-opératoires les plus fréquentes étaient l’épanchement dans la cavité opératoire (2 cas dans le groupe LS, 3 cas dans le groupe LT), et un écoulement biliaire transitoire (1 cas dans le groupe LS). Aucune récidive n’a été observée lors d’un suivi de 6 à 12 mois.

*Conclusion* : La cystectomie par voie laparoscopique semble être efficace et sûre pour le traitement de patients sélectionnés avec des kystes uniques, de petite taille, et superficiels dans le foie. Des recommandations précises concernant cette technique et ses indications ne pourront être données qu’après des études prospectives.

## Introduction

Echinococcosis is a human parasitic disease that has been reported worldwide, especially in the pastoral regions. In many developing countries, echinococcosis has been considered as a major threat to public health [14]. To date, two major types of echinococcosis have been reported in the epidemic regions including cystic echinococcosis (CE) and alveolar echinococcosis (AE), among which CE is the most common type in humans, and the liver the major affected organ [10]. According to the recommendations proposed by WHO, radical surgery is the main strategy for the treatment of hepatic cystic echinococcosis (HCE) [4].

In the past, partial removal of the CE cyst germinal layer and inactivation by protoscolecidal agents were used for the surgical treatment of HCE. The method is minimally invasive and has few surgical risks. However, many postoperative complications have been reported such as postoperative biliary fistula, remnant cavity infections and relapse. Repeated surgeries are needed due to failure of the first surgery, which causes severe health problems and a heavy economic burden for the patients [16]. In the previous decades, endo-cystectomy has been used for the treatment of CE in clinical practice, which reduces the occurrence of postoperative complications [2]. Recently, minimally invasive surgeries have been well accepted by patients. The initial attempt to perform minimally invasive surgery for CE began with endo-cystectomy under laparoscopy (LS) [9]. Although the surgery was minimally invasive, complications were still inevitable. Therefore, total cystectomy using LS has been proposed for treating HCE and is increasingly used in clinical practice [15].

In this retrospective analysis, 22 HCE cases admitted to our department from June 2009 to June 2013 were included to investigate the treatment outcomes and the complications of total cystectomy under LS.

## Patients and methods

### Ethics statement

Written informed consents were obtained from each subject. This study was approved by the Ethics Committee of the First Affiliated Hospital of Xinjiang Medical University.

### Patients

A total of 22 patients (male: 12, female: 10) receiving total cystectomy were enrolled in this retrospective analysis. The eligible patients were those confirmed with HCE from color Doppler ultrasound and computed tomography (CT) with typical images. Prior to the surgery, CE was serologically confirmed in these patients using the Dot Immunogold Filtration Assay (DIGFA) against *Echinococcus* sp. antigens [8]; positive results were obtained in 18 cases and weak positive results were obtained in 4 cases. In addition, the cardiac and pulmonary function of each patient was evaluated to determine whether the patients would tolerate laparoscopic operation. Hepatic CT angiography was performed prior to surgery to assess the location of the hepatic CE cyst and its association with the peripheral vessels. The exclusion criteria were: patients who could not tolerate laparoscopy, and those with cysts closely adjacent to the large blood vessels.

### Preoperative preparation

The patients were in fasting state before surgery. Gastrointestinal decompression was performed for each patient on the morning of surgery. Urinary catheter was inserted for each patient.

### Operation

**Figure 1.**
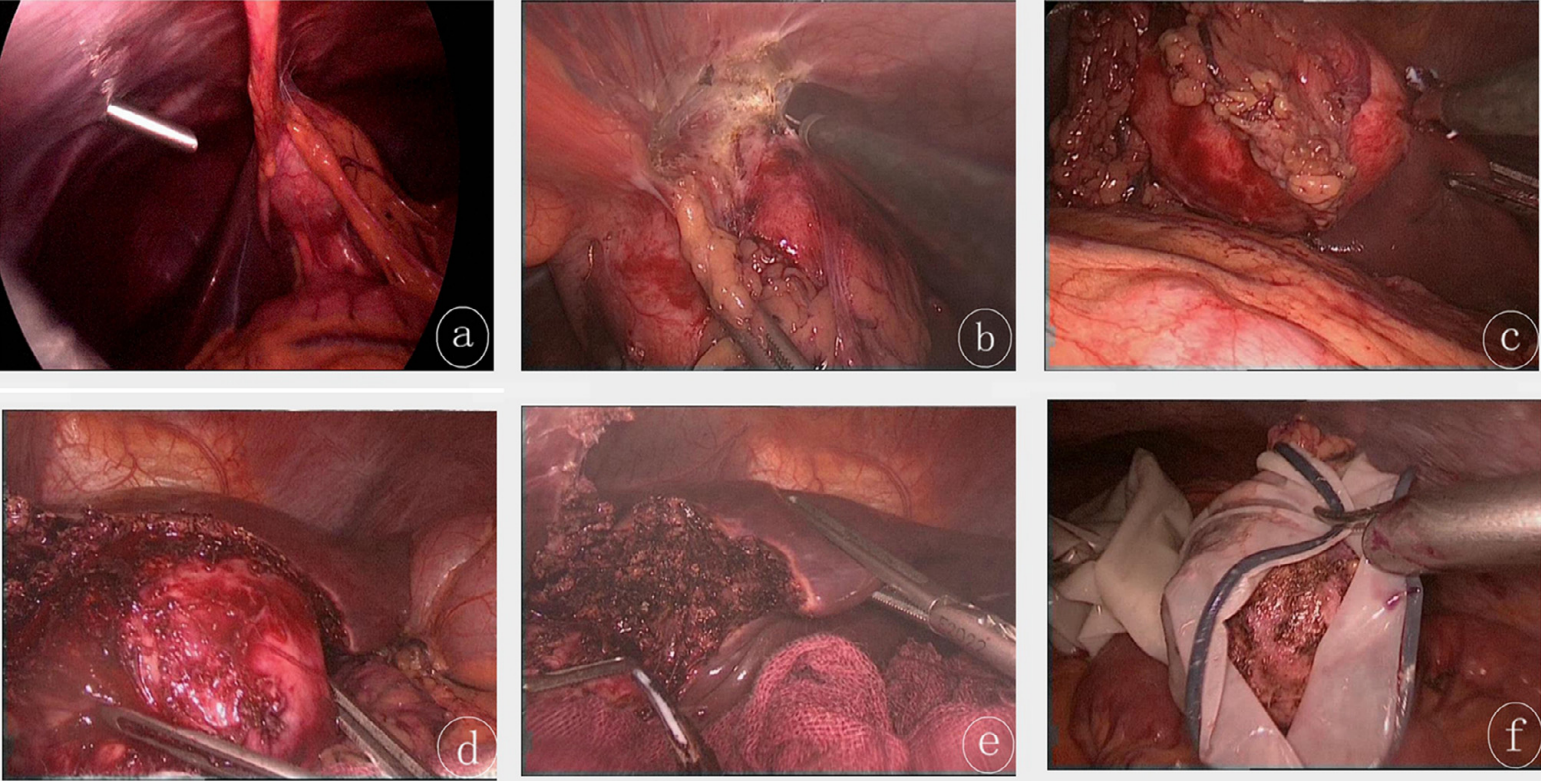
(a) The echinococcosis cyst was located in the left lobe of the liver as revealed by laparoscopy. The cyst was adjacent to the peripheral tissues. (b) Monopolar electrocoagulation was performed to free the cyst from the peripheral tissue. (c) The cyst was separated from the liver using an ultrasound scalpel. (d) The ultrasound scalpel was used to dissociate the cyst and the liver adventitia, by delineating the hepatic interspace between the cyst and the liver. (e) The remaining liver wound after removal of the cyst. (f) The cyst was placed in a surgical bag designed by our team, using a latex medical glove: the front part (also called finger part) of the glove was cut using scissors, and served as the entry to the bag. The opening of the glove was ligated using a suture. The cyst was then removed from the abdomen of the patient.

Prior to the surgery, general anesthesia was performed using tracheal cannula and combined anesthesia (midazolam 0.15 mg/kg; pancuronium bromide 0.2 mg/kg; fentanyl l5–10 μg/kg; via intravenous injection). The patients were in the supine position. Subsequently, CO_2_ pneumoperitoneum was established, and intra-abdominal pressure was maintained in a range of 12–14 mmHg. Afterwards, an inspection hole was made at a position that was above the navel in order to introduce the laparoscope. On this basis, the location of the cyst was evaluated. Once the position of the cyst was localized, three operating holes were made beneath the xiphoid process, on the left/right costal margin along the mid-clavicular line, and on the left anterior axillary line beneath the costal margin, respectively. Surgical procedures were as follows (Fig. 1): Step 1. The cysts were separated from the peripheral tissues and organs; Step 2. Medical gauzes soaked in 10% hypertonic saline solution were used to protect the normal tissues adjacent to cysts; Step 3. The liver capsule was opened using an ultrasound scalpel along the external margins of the cysts. In the presence of significant capillary hemorrhage, immediate hemostasis was carried out using a monopolar electrocoagulation or ultrasound scalpel. Afterwards, the cysts were dissected gradually using an ultrasound scalpel along the potential fibrous capsule formed between the cysts and the liver. Meanwhile, large blood vessels and bile ducts in the external capsule of the cysts were closed using absorbable clips until total cystectomy was completed. Step 4. Monopolar electrocoagulation was used for electrocauterization of the hepatic wound surface. Additional examination was carried out to observe whether active bleeding and/or biliary fistula were present. Local surgical areas were covered using biological absorbable hemostatic gauzes. Step 5. The hydatid cyst was placed into a sampling bag, and then the opening of the bag was extracted out from the abdominal wall through the incision after dilation of the inferior margin of the xiphoid process. Cyst puncture suction was performed in the bag, and the cyst was removed after diminution of its size. Alternatively, the cyst was taken out from an incision made on the abdominal wall. Step 6. A drainage tube was inserted around the surgical region.

## Results

### Patient characteristics

The characteristics of the 22 patients enrolled in this study are summarized in Tables 1 and 2. In total, a laparoscopic approach for total cystectomy was accomplished in 15 patients (male: 8, female: 7) with a mean age of 40.3 years (Table 1). Seven patients (male: 2, female: 5) with a mean age of 38.4 years were transferred to open surgery (Table 2).

**Table 1.** Demographic data for patients who underwent laparoscopic approach for total pericystectomy.

Case	Sex	Age	Lesion of liver			Other diseases	Operation		LOS (day)	Complication	Effect
			Types	Location	Diameter (cm)		Time (min)	Bleeding (ml)			
1	F	31	CE_3_	Left lobe	6 × 4	None	210	100	7	None	Cured
2	M	23	CE_2_	Right lobe	5 × 5	None	180	150	6	None	Cured
3	F	36	CE_3_	Right lobe	4 × 5	None	160	120	7	None	Cured
4	M	68	CE_2_	Right lobe	10 × 5	Gallstone	200	100	8	Hydrops	Cured
5	M	25	CE_2_	Left lobe	9 × 8	None	190	200	6	None	Cured
6	M	33	CE_3_	Left lobe	5 × 4	None	170	80	7	None	Cured
7	M	37	CE_3_	Right lobe	6 × 5	None	180	120	7	None	Cured
8	M	35	CE_3_	Right lobe	5 × 4	None	150	50	6	None	Cured
9	F	64	CE_2_	Right lobe	6 × 5	None	160	80	7	None	Cured
10	F	31	CE_2_	Right lobe	6 × 3	None	150	80	6	None	Cured
11	F	62	CE_3_	Right lobe	4.8 × 4.6	PLG, PCE	170	100	15	Bile leakage	Cured
12	F	56	CE_2_	Left lobe	6.7 × 6	None	200	100	8	None	Cured
13	F	33	CE_3_	Left lobe	6 × 4	Inguinal hernia	160	80	6	None	Cured
14	F	41	CE_2_	Right lobe	6 × 4	None	170	100	8	Hydrops	Cured
15	M	19	CE_1_	Right lobe	6 × 4	None	160	80	6	None	Cured

LOS: length of stay; PLG: polypoid lesion of gallbladder; PCE: pulmonary cystic echinococcosis. Type (WHO/IWGE [4]): CE1-simple cyst; CE2-multiple daughter cyst; CE3-cyst with detachment of membranes.

**Table 2.** Clinical materials of patients who underwent open surgery.

Case	Sex	Age	Lesion of liver			Other diseases	Operation		LOS (day)	Complication	Effect
			Types	Location	Diam (cm)		Time (min)	Bleeding (ml)			
1	F	21	CE_2_	Right lobe	6 × 8	None	190	300	8	None	Cured
2	M	43	CE_2_	Right lobe	7 × 8	PLG	200	350	10	None	Cured
3	F	33	CE_3_	Left lobe	8 × 10	None	150	160	11	Hydrops	Cured
4	M	49	CE_2_	Right lobe	5 × 8	None	180	260	10	None	Cured
5	M	28	CE_2_	Right lobe	6 × 8	None	190	210	9	None	Cured
6	M	30	CE_3_	Right lobe	6 × 10	None	230	340	8	Hydrops	Cured
7	M	65	CE_3_	Right lobe	6 × 9	Cholecystitis	200	240	15	Hydrops	Cured

LOS: length of stay; PLG: polypoid lesion of gallbladder. Type (WHO/IWGE classification [4]): CE_2_-multiple daughter cyst; CE_3_-cyst with detachment of membranes.

### Intraoperative conditions

During the pre-laparoscopic operation, there were some surgical difficulties; seven patients were transferred to open surgery: two patients (9.1%) because of hydatid cyst rupture, three (13.6%) because of poor field exposure induced by massive hemorrhage, and two patients (9.1%) in order to avoid long-term catheter-carrying because biliary fistula repair was necessary. Among the seven patients, six patients received total cystectomy under laparotomy, while the other one received endo-cystectomy and subtotal cystectomy. In total, the procedures lasted for 150–230 min with a mean of 177 min. During the procedures, the hemorrhage volume ranged from 160 mL to 350 mL (mean: 237 mL). Among these patients, six patients received cholecystectomy as the cysts were close to the gallbladder and three patients received biliary tract exploration as biliary fistula was identified.

A laparoscopic approach for total cystectomy was successfully accomplished in 15 patients. During LS surgery, no uncontrollable hepatic hemorrhage was observed. The whole LS surgery lasted for 160–210 min (mean: 174 min) and the hemorrhage volume was about 80–200 mL (mean hemorrhage volume: 103 mL). Moreover, no blood transfusion was required during LS surgery. Concurrent gallbladder lithiasis was noted in one patient, and one patient suffered from gallbladder polyps. On this basis, laparoscopic cholecystectomy was performed in these patients. Additionally, one patient had indirect inguinal hernia, and herniorrhaphy was performed under LS. After cystectomy, the cysts were taken out from a supplementary incision made on the abdominal wall in four patients. For the other patients (*n* = 11), the incision made beneath the xiphoid process was dilated to open the cyst into the sampling bag and the contents of the cyst were removed using an aspirator before extracting the sampling bag from the incision.

### Postoperative conditions

All seven patients transferred to open surgery could walk and eat 3–4 days after surgery. First flatus was noticed in these patients about 2–3 days after open surgery. Postoperative alanine aminotransferase (ALT) and aspartate aminotransferase (AST) levels were 70–248 U/L and 58–245 U/L, respectively, which were 5.2-fold and 5.0-fold higher than the baseline levels. The levels of ALT and AST were in the normal range about 5–7 days after open surgery. Hydrops in the surgical area was noted in three patients. Drainage of the abdominal cavity was maintained for 5–8 days. Wound healing of all the patients was satisfactory with no complications of bile leakage, postoperative hemorrhage and adhesion of intestine, or obstruction. No mortality was reported. The hospitalization lasted for 8–15 days (mean: 10.1 days). To prevent relapse of HCE, albendazole was administered in four patients for an average of 4.6 months (range: 3–6 months), while three patients with open surgery for total cystectomy received no drug treatment. The follow-up duration was 6–12 months. Within this follow-up period, color Doppler ultrasound examination indicated no relapse or peritoneal seeding in these patients.

Fifteen patients who underwent total cystectomy under LS could walk and eat 1–2 days after surgery. First flatus was noticed 1–2 days after the surgery. Postoperative ALT and AST were 80–168 U/L and 78–225 U/L, respectively, which were 4.6-fold and 4.9-fold higher than normal levels, and decreased to normal levels in about 3–5 days after surgery. After surgery, drainage of the abdominal cavity was performed for 3–5 days until no liquid was observed. Hydrops was noticed in one patient and was aspirated immediately. A small amount of bile leakage was present in one patient in the drainage tube the day after surgery. On the fifth day, the bile leakage resolved spontaneously. No hydrops was found in the abdominal cavity. Wound healing in all patients was satisfactory with no complications of postoperative hemorrhage and adhesion of intestine or obstruction. No mortality was reported. The hospitalization lasted for 6–15 days (mean 7.3 days). Among these patients, one patient had pulmonary CE and was admitted to the department of thoracic surgery for removal of a pulmonary cyst under thoracoscopy 6 days later. For these patients, no albendazole was given after total cystectomy. Follow-up was carried out for 6–12 months. Within this follow-up period, no CE relapse or abdominal cavity cyst metastasis was noticed as revealed by color Doppler ultrasound technique.

## Discussion

HCE is a common zoonotic parasitic disease that has been reported worldwide. Generally, the clinical symptoms are vague or occult in patients with early-stage HCE. With the increase in size and/or complications of the cysts, abdominal discomfort or more specific signs and symptoms are noted. In the past decades, the diagnosis of HCE has mainly depended on ultrasound mass screening in endemic regions of China, especially in Xinjiang Autonomous Region. Surgery has been considered as the treatment of choice [3]. Recently, with advances in medical technology, radical surgery, i.e. cystectomy and partial hepatectomy, has been more commonly used in clinical practice, especially total cystectomy. Total cystectomy can eliminate the residual cavity and avoid the discomforts caused by residual cavity hydrops, infections, and bile leakage, and it is the best prevention method against recurrence of the parasitic disease [1]. During the operation, the margins of the external capsule (or “adventitia”) should be accurately identified. Subsequently, cystectomy is performed along this interspace to remove the hydatid cysts completely and reduce hemorrhage from the hepatic wound surface. Total cystectomy under LS is a type of surgery in which the CE cysts are removed through minimally invasive surgery [5, 7, 11]. However, the technique is not commonly used in clinical practice due to limitations of laparoscopic technology and demanding requests for complete cystectomy. Twenty-two patients in our hospital were selected for the LS approach. Among them, seven patients had to be transferred to open surgery and in 15 patients, total cystectomy could be achieved under LS. Our results indicate that the laparoscopic approach for total cystectomy can be performed safely in these patients. Nevertheless, there are still limitations for this type of surgery due to laparoscopic technology, surgeon training, and technical conditions in most endemic regions.

With regard to the laparoscopic approach for total cystectomy, patients with severe cardiac, pulmonary, and renal diseases or systematic diseases should be excluded. Moreover, the patients are required to be tolerant to anesthesia, artificial pneumoperitoneum, and laparoscopic surgery. During the operation, more attention should be paid to the following aspects, including: (i) *Determination of CE cyst size*. In this study, the diameters of the cysts in patients transferred to open surgery were more than 8 cm, a size which posed a major challenge for the exposure of the surgical field and the cystectomy. Two patients with cysts of more than 8 cm in diameter successfully underwent an LS approach for total cystectomy as the main cyst was mostly located outside the liver with small portions adjacent with normal hepatic parenchyma. Usually, a cyst diameter of less than 6 cm is preferable. (ii) *Location of CE cysts.* According to our clinical experience, a higher success rate of operation was found for patients with a smaller connecting area between the cysts and the hepatic surface. If the cyst is located deeper in the hepatic parenchyma, even with small volume, comparatively large residual hepatic wounds are induced, resulting in possible uncontrolled hemorrhage. (iii) *Selection of CE cyst types*. Various degrees of fibrosis were observed in the external wall of the cyst, associated with the length of time during which the cyst coexisted with body organs. Cysts with severe fibrosis were easily dissected with rare risk of abdominal cavity metastasis. Therefore, paradoxically, higher surgery risk was found in patients with CE1 compared with that of CE2 and CE3 cysts. (iv) *Technological equipment of laparoscopes*. The use of ultrasound scalpel contributed considerably to a smoother process of LS surgery by identifying the interspace between the external cyst wall and hepatic tissue. Meanwhile, cyst rupture caused by heat conduction may be avoided, which could reduce the surgical risks. (v) *Exposure of the surgical field*. As the small interspace between HCE and hepatic tissue led to difficult surgical field exposure, additional assistance was necessary for the interspace exposure. Some fibrous adhesions of cyst surface to the surrounding liver may be preserved; in our practice, this contributed to a better exposure of the surgical field. (vi) *Hemorrhage risk during surgery*. During LS surgery, many large vessels were exposed, which were closed by absorbable clips. Slight hemorrhage was controlled by the ultrasound scalpel or electric coagulation. We may expect that the indications of cystectomy would be extended by the use of selective hemi-hepatic blood flow occlusion under the laparoscope [12]. (vii) *Exposure of hepatic ligaments and adhesions around cysts to cyst content*. The surgical field was protected by gauzes with hypertonic saline solution in order to prevent unexpected rupture of the cysts resulting in abdominal cavity seeding of the protoscoleces or germinal cells. (viii) *Extraction of the cyst after removal.* Dissociated cysts were put into a sampling bag and were then taken out. Because of the risk of protoscolex spillage, the bag should be protected in order to avoid any hydatid fluid leakage. The bag was extracted out of the puncture hole created in the abdominal wall for cyst puncture. Part of the hydatid fluid and endocyst debris were aspirated by a suction apparatus and the bag was then taken out to prevent hydatid fluid leakage that could induce regional pollution. An additional hole was made in the abdominal wall to take out the whole CE cyst in some patients, when the risk of peritoneal infection by the cyst content was judged too high if the cyst were to be extracted through the initial hole.

Radical surgery was not performed in three patients: rupture of cysts prevented total cystectomy in two patients, and one patient underwent endo-cystectomy. After these procedures, the remnant cavity was treated using 10% hypertonic saline solution [13], and the patients received albendazole to avoid relapse.

In this study, 15 patients with HCE underwent total cystectomy under LS. Prior to surgery, imaging must be carried out to confirm the diagnosis and type of cyst, using the WHO-IWGE classification [4], and assess the location and size of the lesion as well as the relations between the cysts and the hepatic vessels and bile ducts. On this basis, it is possible to choose suitable surgery according to international recommendations [4, 6]. Laparoscopy is required first to determine whether total cystectomy may be completely performed using this approach. Otherwise, patients should be transferred to open surgery. Although 15 patients successfully underwent total cystectomy with small amounts of hemorrhage, short-term hospitalization, and few complications as well as satisfactory healing, we may consider that this surgery is not mature. Additional questions such as the indication of complementary albendazole treatment and its length before and after the LS surgical procedure still remain open. Long-term follow-up is also necessary to confirm the absence of recurrence observed after the rather short-term follow-up of 6–12 months we had for these patients. Therefore, further studies are needed to investigate the effectiveness of this technique for the treatment of CE, using prospective, randomly controlled trials.

In conclusion, this retrospective study of the laparoscopic approach for total cystectomy shows that it is effective in treating hepatic CE in selected patients. It can eliminate the whole echinococcosis lesion with minimal wound. It appears to be a safe and effective method, which could be further used in clinical practice. Specific recommendations for its indication and technical aspects would benefit from prospective studies.
